# Isolated metachronous splenic metastasis from synchronous colon cancer

**DOI:** 10.1186/1477-7819-4-42

**Published:** 2006-07-06

**Authors:** Rasim Gencosmanoglu, Fugen Aker, Gozde Kir, Nurdan Tozun

**Affiliations:** 1 Department of General Surgery, Marmara University School of Medicine, Istanbul, Turkey; 2 Unit of Surgery, Marmara University Institute of Gastroenterology, Istanbul, Turkey; 3 Department of Pathology, Haydarpasa State Hospital, Istanbul, Turkey; 4 Department of Pathology, Umraniye State Hospital, Istanbul, Turkey; 5 Department of Gastroenterology, Marmara University School of Medicine, Istanbul, Turkey

## Abstract

**Background:**

Isolated splenic metastases from colorectal cancer are very rare and there are only 13 cases reported in the English literature so far. Most cases are asymptomatic and the diagnosis is usually made by imaging studies during the evaluation of rising CEA level postoperatively.

**Case presentation:**

A 76-year-old man underwent an extended left hemicolectomy for synchronous colon cancers located at the left flexure and the sigmoid colon. The tumors were staged as IIIC (T3N2M0) clinically and the patient received adjuvant chemotherapy. During the first year follow-up period, the patient remained asymptomatic with normal levels of laboratory tests including CEA measurement. However, a gradually rising CEA level after the 14^th ^postoperative month necessitated further imaging studies including computed tomography of the abdomen which revealed a mass in the spleen that was subsequently confirmed by ^18^FDG- PET scanning to be an isolated metastasis. The patient underwent splenectomy 17 months after his previous cancer surgery. Histological diagnosis confirmed a metastatic adenocarcinoma with no capsule invasion. After an uneventful postoperative period, the patient has been symptom-free during the one-year of follow-up with normal blood CEA levels, although he did not accept to receive any further adjuvant therapy. To the best of our knowledge, this 14^th ^case of isolated splenic metastasis from colorectal carcinoma is also the first reported case of splenic metastasis demonstrated preoperatively by ^18^FDG PET-CT fusion scanning which revealed its solitary nature as well.

**Conclusion:**

Isolated splenic metastasis is a rare finding in the follow-up of colorectal cancer patients and long-term survival can be achieved with splenectomy.

## Background

Splenic metastases from colorectal carcinoma are very rare and usually occur as a component of disseminated disease. From the first case report of isolated metastasis to the spleen in the setting of colorectal carcinoma by Dunbar *et al*. [1] in 1969, only thirteen cases are reported in the English literature to date [2-13]. Twelve of them were metachronous metastases, whereas the remaining one synchronous. Although the exact incidence of splenic metastases is still unknown, Berge [14] reported the overall incidence of splenic involvement as 7.1% in 7165 autopsy cases with various cancers and the incidence of splenic metastasis from colon and rectal carcinomas as 4.4% and 1.6%, respectively. However, he did not report any case of solitary splenic metastasis. Anatomical, histological and functional features of spleen have been speculated as the reason of the rarity for solitary cancer metastasis [15]. Most cases are reported as asymptomatic and the diagnosis is usually made by the imaging studies such as abdominal ultrasound (US) or computerized tomography (CT) during the evaluation of a rising CEA level in the postoperative follow-up period of colorectal cancer patients. To the best of our knowledge, this is the first case in which the splenic metastasis from colorectal carcinoma was demonstrated preoperatively by ^18^FDG PET-CT fusion scanning which also revealed its solitary nature.

## Case presentation

**Figure 1:**
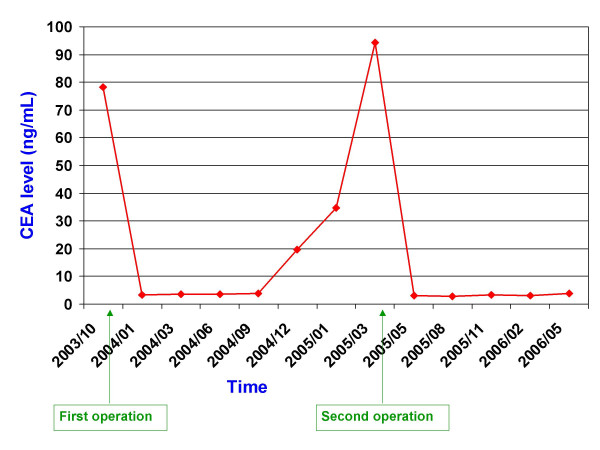
The course of pre- and post-operative blood CEA levels (Reference values: 0–10 ng/mL).

**Figure 2:**
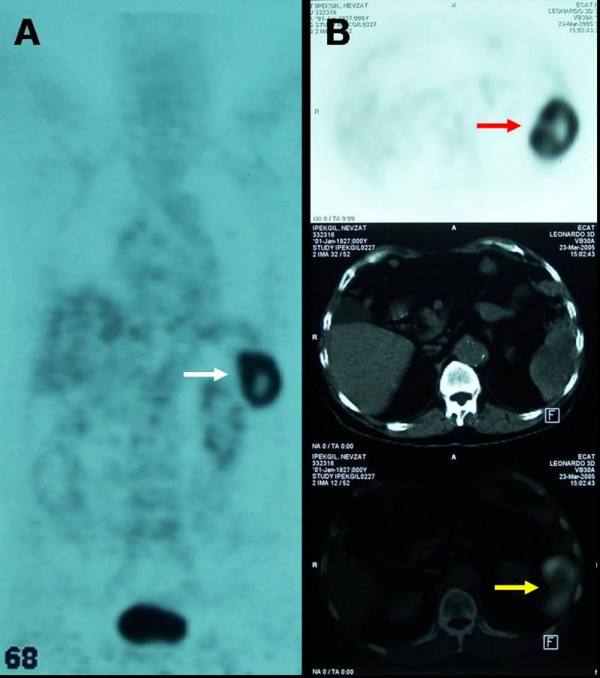
^18^FDG- PET scanning showed an isolated hypermetabolic state in the spleen (white arrow) (A). PET-CT fusion scanning confirmed that this pathological FDG uptake (red arrow) superimposed with the hypodense mass in the spleen (yellow arrow) (B).

**Figure 3:**
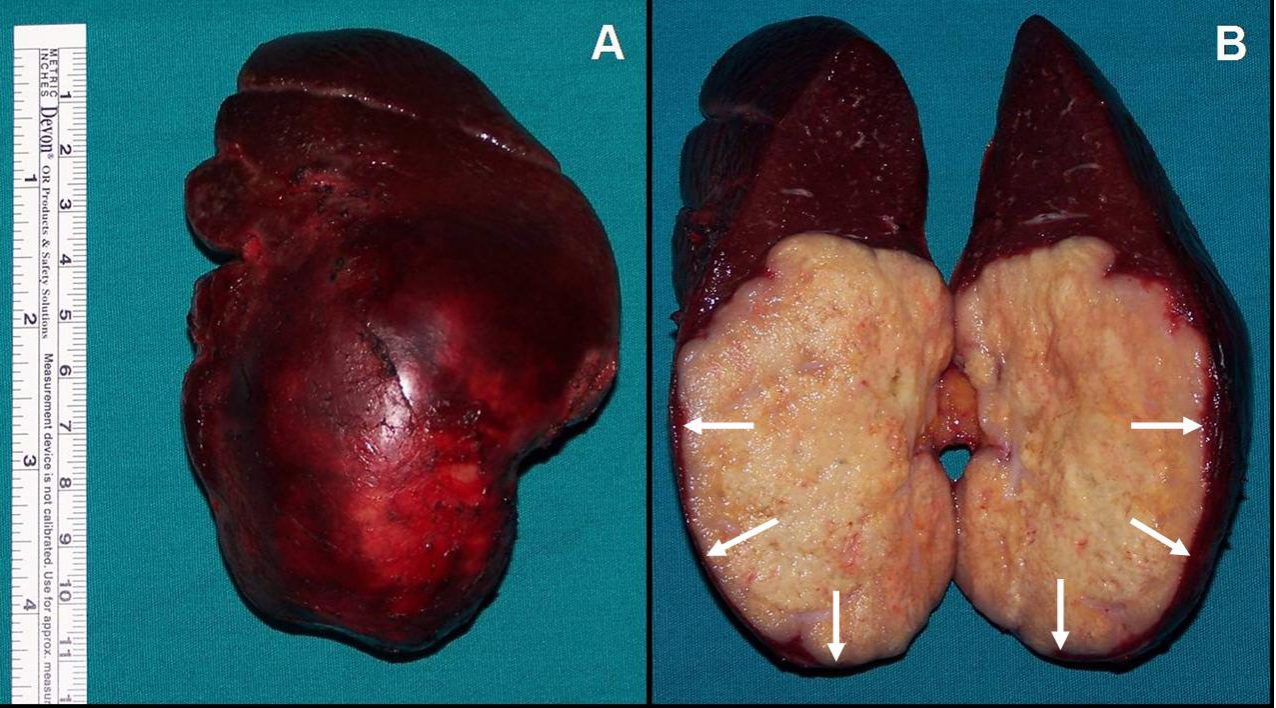
The macroscopic view of the splenectomy specimen (A) and its cross section (B). Note that the splenic capsule was not invaded by the metastatic tumor (arrows).

**Figure 4:**
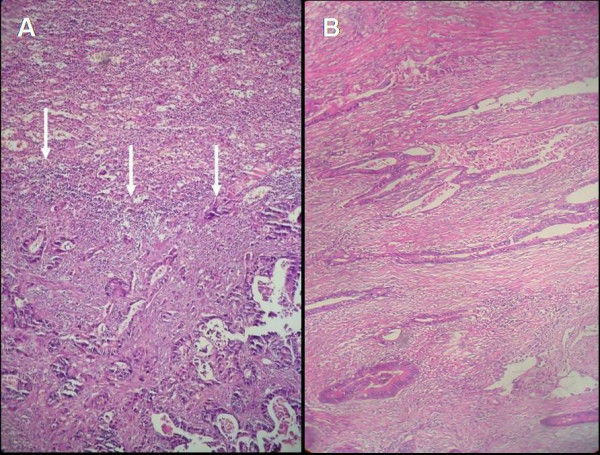
Histological section of the splenic tumor showing glandular pattern consistent with metastasis from colonic adenocarcinoma (arrows show the tumor within the splenic parenchyma), X100, Hematoxylin +Eosin (A). Histologic section of the primary colonic tumor located at the sigmoid colon with glandular pattern, X100, Hematoxylin +Eosin (B).

A 76-year-old man suffering from fatigue, weight loss and rectal bleeding underwent extended left hemicolectomy with the diagnosis of synchronous colon cancer. Preoperative work-up including colonoscopy and contrast-enhanced abdominal computed tomography (CT) showed that the tumors located at the left flexure and the sigmoid colon and the endoscopic biopsies revealed the diagnosis of adenocarcinoma for both of the tumors. There was neither liver nor other remote site metastasis in the abdomen. The chest X-ray was normal. However, CEA level was higher (34.63 ng/mL, Reference values: 0–10 ng/mL) in the preoperative period (Figure 1). Histopathological examination of the specimen revealed a moderately differentiated adenocarcinoma, 2 cm in diameter, that invaded into but not beyond the muscularis propria (pT2) at the left flexure with lymphatic and vascular but not perineural invasion and another moderately differentiated adenocarcinoma, 6 cm in diameter, that invaded into the serosa (pT3) with lymphatic, vascular and perineural invasion. Both of the tumors had similar histologic features that were well differentiated and complex glands composed of stratified columnar cells with eosinophilic cytoplasms accompanied by large necrotic areas. Four of the nine lymph nodes were positive for metastatic adenocarcinoma with the presence of perinodal invasion (pN2). Therefore, the patient was classified as stage C2 according to the modified Astler-Coller staging system and stage IIIC (pT3N2, cT3N2M0) according to the TNM system. He received oral chemotherapy consisting of capecitabine (Xeloda^®^, Roche) 5 × 500 mg capsule for 14 days with 7-day interval for a total of 8 courses. Blood CEA level decreased to the 3.54 ng/mL in the early postoperative period and remained normal up to the 14^th ^month of follow-up. A rising CEA level (Figure 1) in this asymptomatic case necessitated further imaging studies including abdominal ultrasound and contrast-enhanced abdominal and thoracic CT which revealed a hyperdense solid mass in the lower two third of the spleen, while the liver and the paraaortic area were disease-free. Furthermore, ^18^FDG- PET scanning showed an isolated hypermetabolic state in the spleen (Figure 2A), while PET-CT fusion scanning confirmed this pathological FDG uptake as superimposing with the hypodense mass in the spleen (Figure 2B). Any other remote organ metastases were not found by both CT and PET scanning. Following a preoperative vaccination against *Streptococcus pneumoniae*, *Haemophilus influenzae*, and *Neisseria meningitidis *as prevention strategy to avoid overwhelming postsplenectomy infection, the patient underwent splenectomy 17 months after his previous colorectal cancer surgery. At laparotomy, the tumor was in the lower two third of the spleen without any capsule invasion macroscopically (Figure 3) and there were neither liver nor other intraabdominal organ metastases. Also, any lymph node involvement at splenic hilus or paraaortic site was not detected during the operation. Therefore, lymphadenectomy at either site was not performed. Histopathologic examination of the splenectomy specimen showed that the tumor within the spleen was a metastasis of a moderately differentiated adenocarcinoma with 5 × 6 × 6.5 cm in dimensions and it was sharply demarcated from the adjacent splenic parenchyma without any capsule invasion (Figure 4A). The histologic features of this metastatic tumor were very similar to the primary synchronous colonic adenocarcinomas (Figure 4B). Because the patient had a previous colon cancer surgery and no findings of any primary tumor (i.e. adenocarcinoma) in any other organ except metastatic splenic tumor at laparotomy as well as on the preoperative CT and PET scanning besides the decrease of the high blood CEA level to within normal limits following splenectomy and the presence of histologic similarities between the metastatic tumor and the primaries, the present case was accepted as a solitary metachronous splenic metastasis from colon cancer. The postoperative period was uneventful. The patient has been symptom-free during the one-year of follow-up with a normal blood CEA levels, although he did not accept to receive any further adjuvant therapy.

## Discussion

Several theories have been postulated for the reason of the rarity of solitary splenic metastasis from colorectal cancer. Although the incidence of micrometastases in the spleen secondary to colorectal carcinoma is reported as 4.4% in careful autopsy examinations [14], the rare occurrence of clinically apparent metastasis may propose the existence of a certain mechanism prohibiting tumor cell proliferation in the spleen. The sharp angle of the splenic artery with the celiac axis and the rhythmic contraction by the sinusoidal splenic architecture were speculated as limiting factors of metastasis [9]. Spleen is the second largest organ of the reticuloendothelial system with profuse monocytes, immunoglobulin synthesis, and opsonin production. Therefore, immune surveillance appears to potently inhibit tumor cell proliferation [9]. On the other hand, intense destruction of malignant cells in spleen may limit the development of splenic metastasis as supported by the experimental evidences [16]. The absence of afferent lymphatics to the spleen is considered to be another reason for such low incidence of splenic metastasis [12].

Another considerable point is the potential relationship between the primary tumor site and isolated splenic metastasis. In fact, in 12 of the 14 reported patients including the present case, the primary tumor was in the left colon (5 in sigmoid colon, 3 in descending colon, 2 in rectum, and 2 at left flexure), whereas it was in cecum and ascending colon in the remaining two. As previously stated by Avesani *et al*. [11], this observation may support the theory by Indudhara *et al*. [6]; a possible retrograde spread to the spleen via the inferior mesenteric vein. However, this explanation has to stay theoretical; because, there is unfortunately no current imaging study to be able to show this type spread in an individual basis.

Diagnosis of splenic metastasis as well as other remote organ metastasis from colorectal cancer can be made by help of many current imaging studies such as US, CT or MRI. However, FDG-PET scanning provides a better detection of metastatic deposits, especially when they are too small to be shown by other radiological studies in the setting of postoperatively rising CEA level. In addition, PET-CT fusion may specifically suggest that the mass seen by the CT is tumoral in nature. On the other hand, solitary status of metastasis can be demonstrated and predicted preoperatively by PET-CT fusion scanning as in the present case. Therefore, the indication of splenectomy can be justified in such cases since the presence of multiple remote site metastases usually exterminates the chance of surgical intervention.

Long-term survival rate following splenectomy in patients with solitary splenic metastasis from colorectal cancer is still unknown. However, the limited data extracted from the case reports in the literature indicate that these patients may survive up to 7 years [8,12,13]. The present case is disease-free during the one-year of follow up.

## Conclusion

Isolated splenic metastasis is a rare finding in the follow-up of colorectal cancer patients and long-term survival can be achieved with splenectomy.

## Abbreviations

CEA: carcinoembryonic antigen

^18^FDG-PET: ^18^Fluorodeoxyglucose – positron emission tomography

PET-CT: positron emission tomography – computed tomography

US: ultrasound

CT: computed tomography

MRI: magnetic resonance imaging

## Competing interests

The author(s) declare that they have no competing interests.

## Authors' contributions

**RG **performed the operations and prepared the manuscript. **FA **and **GK **performed the histopathologic evaluations. **NT **participated in the redaction of the article. All authors read and approved the final manuscript.
